# Prevalence of neutropenia among adult Arabs in Qatar: Relation to other hematological parameters and anthropometric data

**DOI:** 10.1097/MD.0000000000030431

**Published:** 2022-09-09

**Authors:** Mohamed A. Yassin, Ashraf T. Soliman, Saloua M. Hmissi, Mohammad A.J. Abdulla, Maya Itani, Ans A. Alamami, Mahmood B. Aldapt, Aasir M. Suliman, Ezzeddin A. Ibrahim, Mouhand F.H. Mohamed, Waail Rozi, Shehab F. Mohamed, Prem Chandra, Abdulqadir J. Nashwan

**Affiliations:** a Department of Medical Oncology/Hematology, National Centre for Cancer Care and Research (NCCCR), Hamad Medical Corporation (HMC), Doha, Qatar; b Division of Endocrinology, Department of Pediatrics, Hamad General Hospital (HGH), Hamad Medical Corporation (HMC), Doha, Qatar; c Blood Transfusion Center, Hamad Medical Corporation (HMC), Doha, Qatar; d Department of Dietetics and Nutrition, Hamad Medical Corporation (HMC), Doha, Qatar; e Department of Critical Care Medicine, Hamad General Hospital (HGH), Hamad Medical Corporation (HMC), Doha, Qatar; f Department of Internal Medicine, Hamad Medical Corporation (HMC), Doha, Qatar; g Medical Research Center, Hamad Medical Corporation (HMC), Doha, Qatar; h Department of Nursing, Hazm Mebaireek General Hospital (HMGH), Hamad Medical Corporation (HMC), Doha, Qatar.

**Keywords:** absolute neutrophil count, complete blood count, ethnicity, neutropenia, reference range

## Abstract

Neutropenia ranges from a normal variant to life-threatening acquired and congenital disorders. This study aims at providing baseline information regarding the prevalence and spectrum of neutropenia in the Arab blood donors who are living in Qatar. This retrospective cohort study was conducted to review the data of healthy Arab individuals (≥18 years) who donated blood between January 1, 2015 to May 15, 2019. A complete blood count was performed using automated analyzers. The prevalence of neutropenia was 10.7%. The prevalence in females was 32% and in males, it was 6%. Absolute neutrophil count (ANC) below 1 × 10^9^/L was detected in 10% of Arab females and 1.8 % of Arab males. In females, the neutropenic group had significantly lower hemoglobin (Hb) levels and higher red cell distribution width, and lower total white blood cells and lymphocyte counts (*P* < .001) compared to the group with ANC > 1.5 × 10^9^/L. Significant correlations were found between the ANC and Hb (*r* = 0.33, *P* < .05) and ANC and total white blood cells (*r* = 0.45, *P* < .01). The prevalence of neutropenia is considerably high in Arab adult females compared to other ethnic groups. Besides the genetic constitution of Arabs, the lower Hb and higher red cell distribution width in females suggest that iron deficiency could contribute to the development of neutropenia.

## 1. Introduction

White blood cells (WBCs), often known as leukocytes, are a diverse category of nucleated blood circulating cells. Their normal reference ranges from 4 × 10^9^/L to 1.1 × 10^10^/L, or 4000 to 11,000 cells per microliter. They are essential for immunity and phagocytosis. The number of WBCs circulating in the blood is measured by a WBCs count, and the proportion of each type of WBC is determined by a WBCs differential. The WBCs differential may also uncover immature cells and potentially life-threatening diseases.^[1–4]^

Only the circulating neutrophils are accounted for in the WBCs count. The half-life of mature neutrophils is about 7 hours. They irreversibly traverse the vascular endothelium into the tissues, where they die after 1 or 2 days.^[1,5]^

Neutropenia can be described as transient (or “acute”) or chronic, extrinsic or intrinsic. Patients with an ANC of 0.2 × 10^9^/L or less virtually have to be admitted to the hospital for intravenous antibiotics. When fever is accompanied by stomach discomfort, antibiotic treatment should include anaerobic coverage. To prevent invasive fungal infection, neutropenic patients should avoid highly polluted sources such as mulch, debris, and bird or animal dung. For many individuals with idiopathic, autoimmune, or cyclic neutropenia, a G-CSF dosage adequate to maintain blood neutrophils higher than roughly 1.0 × 10^9^/L is sufficient.^[3]^

Reference ranges for differential WBCs count: lymphocytes – 1000 to 4000 per mm^3^ (20%–40%), neutrophils – 1500 to 8000 per mm^3^ (55%–70%), monocytes – 100 to 700 per mm^3^ (2%–8%), eosinophils – 50 to 500 per mm^3^ (1%–4%), and basophils – 25 to 100 per mm^3^ (0.5%–1%).^[6]^

Neutropenia is defined as an absolute neutrophil count (ANC) below 1.5 × 10^9^/L (1500/mm^3^), which ranges from a normal variant to life-threatening acquired and congenital disorders. Typically, the severity of neutropenia will determine the functional complications. For example, ANC of 1.0 to 1.5 may warrant investigation of the underlying cause but does not impair the host defense. ANC of 0.5 to 1.0 may slightly increase the risk of infections, but only if other immune system arms are impaired. ANC of 0.2 to 0.5 is associated with an increased risk of infections in most patients. ANC of 0.2 or less (agranulocytosis) carries a risk of severe, life-threatening infections. ANC < 1.5 × 10^9^/L lasting for more than 3 months is usually referred as “chronic neutropenia.”^[1,3,5,6]^

ANC in healthy Asians and Caucasians ranges between 1.5 and 7.0 × 10^9^/L. Individuals of African descent may have ANC < 1.5, as reported in a survey from the US where about 4.5% of black participants had ANC < 1.5. However, it was hypothesized that the neutropenia was attributed to the fluctuation of neutrophil levels cyclically in healthy individuals, where the available evidence now indicates that the fluctuation might not fully explain this variance.^[7–9]^

Still, it is common for healthy individuals to have an occasional ANC 1.5 to 2.0 × 10^9^/L or even lower, especially with the sample obtained early in the day. In this case, periodic complete blood count (CBC) is recommended for their initial evaluation as a baseline.^[10]^

Therefore, this study aims at providing baseline information regarding the prevalence and spectrum of neutropenia in large random samples (n = 600) of Arab blood donors (200 females and 400 males) living in Qatar.

## 2. Materials and Methods

This retrospective cohort study was conducted to review the data of Arab healthy individuals (18 years and above) who donated blood between January 1, 2015 to May 15, 2019 at the Blood Transfusion Center in Qatar. CBC was performed as a part of the blood donation and processed using automated analyzers. Data were collected directly from the patients’ electronic health records. Sample size was estimated following the annual number of healthy blood donators.

### 2.1. Laboratory analysis

The blood specimen was collected in an K2EDTA tube and the CBC was analyzed using the Abbott’s CELL-DYN Sapphire Hematology Analyzer (CD-Sapphire). The hospital central laboratory is accredited by the American College of Pathologists and Joint Commission International. The laboratory met the analytical standards for CBC where the SDI of WBCs count varied from −0.77 to 1.33 from the target mean value during the study period.

### 2.2. Statistical analysis

Data were analyzed using Microsoft Excel 2010 (Microsoft, Redmond, WA) for Windows. All appropriate statistical methods such as independent samples *t*-tests, linear regression, Pearson’s, and Spearman’s correlation coefficient were used. In addition, the power transform (Box–Cox) was used to define the normal range limits of cell counts, which were determined by the mean ± 2 standard deviation. The statistical significance was determined by .05.

## 3. Results

A total of 600 participants were included in the final analysis. The prevalence of neutropenia (ANC < 1.5 × 10^9^/L) in 600 blood donors (200 females and 400 males) was 10.7%. The prevalence in females was 32%, and in males, it was 6%. ANC < 1 × 10^9^/L was detected in 10% of Arab females and 1.8% of Arab males. The prevalence of mild and moderate neutropenia was significantly higher in healthy adult Arab females compared with males.

In males, the neutropenic group had significantly lower hemoglobin (Hb) level and hematocrit value and lower total WBC, lymphocyte, monocyte, and basophil counts (*P* < .001) (Table 1). In females, the neutropenic group had significantly lower Hb level and higher red cell distribution width and lower total WBC, monocyte, and lymphocyte counts (*P* < .001) compared to the group with ANC > 1.5 × 10^9^/L versus ANC > 1.5 × 10^9^/L (Table 2).

**Table 1:** Comparison between males with low ANC (<1.5 × 10^9^/L) versus those with normal ANC (>1.5 × 10^9^/L).

	Age	Height	Weight	Hb	PLT	HCT	MCV	MCHC	RDW	WBC	ANC	LYMP	MONO	ESINO	BASO
M ANC < 1.5	yr	cm	kg	g/dL	×10^9^/L					×10^9^/L	×10^9^/L	×10^9^/L	×10^9^/L	×10^9^/L	×10^9^/L
Mean	34.97	170.79	86.34	13.64	222.59	41.14	80.71	32.27	13.56	4.09	1.01	2.23	0.35	0.13	0.02
SD	12.03	6.67	16.62	2.82	58.79	7.25	5.95	1.62	4.16	2.69	0.24	1.87	0.10	0.10	0.02
CI		2.53	5.79	0.95	19.58	2.42	1.98	0.55	1.43	0.89	0.08	0.63	0.03	0.03	0.01
Upper		178.72	94.12	14.81	247.06	44.57	84.68	33.64	15.27	4.97	1.12	2.91	0.39	0.17	0.03
Lower		176.19	88.33	13.86	227.47	42.15	82.69	33.09	13.84	4.07	1.04	2.28	0.36	0.13	0.02
	**Age**	**Height**	**Weight**	**Hb**	**PLT**	**HCT**	**MCV**	**MCHC**	**RDW**	**WBC**	**ANC**	**LYMP**	**MONO**	**ESINO**	**BASO**
M ANC > 1.5	yr	cm	kg	g/dL	×10^9^/L					×10^9^/L	×10^9^/L	×10^9^/L	×10^9^/L	×10^9^/L	×10^9^/L
Mean	34.77	174.13	90.84	14.54	249.20	44.21	83.75	32.74	15.84	7.01	4.22	3.78	0.57	0.18	0.13
SD	11.44	7.48	18.92	2.00	59.77	3.47	7.42	2.45	6.60	2.41	1.94	7.62	0.31	0.14	2.17
CI		0.65	1.71	0.16	4.93	0.29	0.61	0.20	0.55	0.20	0.96	0.63	0.03	0.01	0.18
Upper		174.97	92.38	14.71	254.13	44.50	84.36	32.94	16.39	7.21	5.18	4.41	0.60	0.19	0.31
Lower		173.67	88.96	14.38	244.27	43.93	83.14	32.54	15.30	6.81	3.25	3.15	0.55	0.17	0.04
*P*-value		.0182	.1576	.0102	.0080	.0001	.0.137	.2506	.0412	.0001	.0001	.2177	.0001	.0331	.7581

ANC = absolute neutrophil count, BASO = basophils, CI = confidence interval, ESINO = eosinophils, Hb = hemoglobin, HCT = hematocrit, LYMP = lymphocytes, MCHC = mean corpuscular hemoglobin concentration, MCV = mean corpuscular volume, MONO = monocytes, PLT = platelets, RDW = red cell distribution width, SD = standard deviation, WBC = white blood cells.

**Table 2:** Comparison between females with low ANC (<1.5 × 10^9^/L) versus those with normal ANC (>1.5 × 10^9^/L).

	Age	Height	Weight	Hb	PLT	HCT	MCV	MCHC	RDW	WBC	ANC	LYMP	MONO	ESINO	BASO
F-ANC < 1.5		cm	kg	g/dL	×10^9^/L					×10^9^/L	×10^9^/L	×10^9^/L	×10^9^/L	×10^9^/L	×10^9^/L
Mean	37.97	158.70	72.90	9.30	243.40	37.34	83.65	32.58	16.86	6.03	1.07	1.68	0.31	0.14	0.02
SD	11.31	6.43	17.26	4.23	65.82	3.50	7.01	1.19	17.70	4.20	0.27	0.41	0.10	0.11	0.01
CI		2.00	5.38	1.32	20.50	1.09	2.18	0.37	5.51	1.31	0.08	0.13	0.03	0.04	0.00
Upper		160.70	78.27	10.61	263.89	38.43	85.84	32.95	22.37	8.99	1.15	1.81	0.34	0.18	0.03
Lower		156.69	67.52	7.98	222.90	36.26	81.47	32.21	11.35	6.37	0.98	1.55	0.28	0.11	0.02
	**Age**	**Height**	**Weight**	**Hb**	**PLT**	**HCT**	**MCV**	**MCHC**	**RDW**	**WBC**	**ANC**	**LYMP**	**MONO**	**ESINO**	**BASO**
F-ANC > 1.5															
Mean	37.97	158.97	75.59	11.97	262.71	38.57	83.95	32.86	14.56	7.68	3.47	2.06	0.45	0.16	0.04
SD	12.33	6.63	16.08	2.78	58.04	2.87	10.96	1.60	3.71	4.99	1.90	0.60	0.22	0.15	0.03
CI		1.51	3.53	0.60	12.44	0.61	2.32	0.34	0.79	1.07	0.41	0.13	0.05	0.03	0.01
Upper		160.48	79.13	12.56	275.15	39.18	86.27	33.20	15.35	8.75	3.88	2.19	0.50	0.20	0.05
Lower		157.45	72.06	11.37	250.27	37.96	81.62	32.52	13.78	6.61	3.06	1.94	0.40	0.13	0.03
*P* value		.8292	.3869	.0001	.0903	.3380	.8701	.3102	.2410	.0644	.0001	.0003	.0001	.4377	.0001

ANC = absolute neutrophil count, BASO = basophils, CI = confidence interval, ESINO = eosinophils, Hb = hemoglobin, HCT = hematocrit, LYMP = lymphocytes, MCHC = mean corpuscular hemoglobin concentration, MCV = mean corpuscular volume, MONO = monocytes, PLT = platelets, RDW = red cell distribution width, SD = standard deviation, WBC = white blood cells.

African Arab males had significantly lower ANC and WBC counts than Asian Arab males (Table 3). The ANC count did not differ significantly between African and Asian Arab females. African females had significantly lower WBC counts and higher Hb concentrations than Asian Arab females (Table 4).

**Table 3:** Comparison between Asian males and African males.

	Age	Height (cm)	Weight (kg)	HB (g/dL)	PLT (×10^9^/L)	HCT	MCV	MCHC	RDW	WBC (×10^9^/L)	ANC (×10^9^/L)	LYMP (×10^9^/L)	MONO (×10^9^/L)	ESINO (×10^9^/L)	BASO (×10^9^/L)
Asian males															
Mean	35.41	173.628	89.752	14.547	246.897	44.483	83.674	32.781	15.630	7.155	3.769	3.405	0.582	0.192	0.041
SD	12.64	7.704	18.661	2.248	60.410	3.380	8.265	2.497	6.366	2.629	2.128	3.513	0.316	0.146	0.046
CI.		0.937	2.077	0.225	6.045	0.339	0.828	0.250	0.638	0.263	0.214	0.352	0.032	0.015	0.006
Upper		174.552	91.890	14.770	252.945	44.821	84.511	33.031	16.268	7.422	3.985	3.757	0.614	0.207	0.048
Lower		172.678	87.737	14.320	240.854	44.143	82.855	32.531	14.993	6.897	3.558	3.054	0.550	0.178	0.037
African males															
Mean	33.65	175.457	92.293	14.429	249.528	43.443	83.697	32.727	15.886	6.240	3.202	3.510	0.515	0.153	0.040
SD	8.93	6.816	18.940	1.700	59.198	4.593	5.313	2.244	6.742	2.232	1.926	3.579	0.268	0.121	0.036
CI		1.122	2.681	0.228	7.921	0.614	6.499	0.300	0.902	0.299	0.258	0.479	0.036	0.016	0.005
Upper		176.565	94.472	14.657	257.448	44.058	93.493	33.027	16.788	6.539	3.460	3.989	0.551	0.169	0.045
Lower		174.321	89.110	14.201	241.607	42.829	80.495	32.427	14.984	5.942	2.944	3.031	0.479	0.136	0.035
*P* value		.0170	.1522	.5020	.6050	.0016	.9706	.7917	.6429	.0001	.0013	.7264	.6092	.0001	.7825

ANC = absolute neutrophil count, BASO = basophils, CI = confidence interval, ESINO = eosinophils, Hb = hemoglobin, HCT = hematocrit, LYMP = lymphocytes, MCHC = mean corpuscular hemoglobin concentration, MCV = mean corpuscular volume, MONO = monocytes, PLT = platelets, RDW = red cell distribution width, SD = standard deviation, WBC = white blood cells.

**Table 4:** Comparison between Asian females and African females.

	Age (yr)	Height (cm)	Weight (kg)	ANC (×10^9^/L)	Hb (g/dL)	WBC (×10^9^/L)	PLT (×10^9^/L)	HCT	MCV	MCHC	RDW	LYMP (×10^9^/L)	MONO (×10^9^/L)	ESINO (×10^9^/L)	BASO (×10^9^/L)
Asian females															
Mean	36.65	159.98	68.05	2.50	11.86	6.76	242.10	37.19	83.04	32.75	14.05	2.09	0.45	0.12	0.03
SD	8.34	5.68	13.55	1.75	2.03	4.35	51.09	3.88	13.21	1.34	1.53	0.68	0.26	0.08	0.02
CI	3.61	2.66	6.01	0.79	0.90	1.98	23.25	1.68	5.71	0.58	0.66	0.29	0.11	0.04	0.01
Upper	40.26	162.64	74.06	3.29	12.76	8.74	265.35	38.87	88.75	33.33	14.71	2.38	0.56	0.16	0.04
Lower	33.04	157.32	62.04	1.70	10.96	4.78	218.84	35.51	77.33	32.17	13.39	1.80	0.34	0.08	0.02
African females															
Mean	39.81	158.65	76.08	2.60	10.92	7.20	259.06	38.38	84.02	32.78	14.14	1.91	0.40	0.17	0.04
SD	12.82	6.76	16.91	1.67	3.78	4.93	63.31	2.96	9.10	1.53	2.28	0.55	0.19	0.15	0.03
CI	2.42	1.34	3.29	0.32	0.72	0.94	12.02	0.56	1.73	0.29	2.22	0.10	0.04	0.03	0.01
Upper	42.23	159.99	79.36	2.92	11.64	8.14	271.08	38.94	85.75	33.07	17.80	2.01	0.43	0.19	0.04
Lower	37.39	157.31	72.79	2.29	10.20	6.27	247.05	37.82	82.29	32.48	13.36	1.80	0.36	0.14	0.03
*P* value	.2600	.4122	.0389	.8035	.2704	.7037	.2498	.1005	.6674	.9307	.8569	.1742	.2864	.1237	.1294

ANC = absolute neutrophil count, BASO = basophils, CI = confidence interval, ESINO = eosinophils, Hb = hemoglobin, HCT = hematocrit, LYMP = lymphocytes, MCHC = mean corpuscular hemoglobin concentration, MCV = mean corpuscular volume, MONO = monocytes, PLT = platelets, RDW = red cell distribution width, SD = standard deviation, WBC = white blood cells.

**Figure 1.**
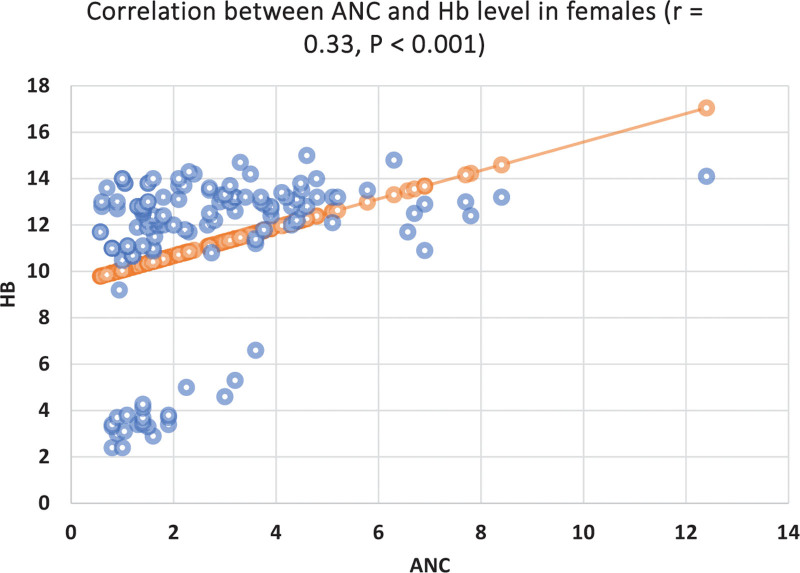
Correlation between Hb level and ANC in females. ANC = absolute neutrophil count, Hb = hemoglobin.

Thirty percent (30%) of the females had Hb < 11 g/dL. Seventy percent of these anemic females had ANC < 1.5 × 10^9^/L. Twenty-four percent of the females with normal Hb level (Hb > 11 g) had ANC < 1.5 × 10^9^/L. Significant correlations were found between the ANC and Hb (*r* = 0.33, *P* < .05) and ANC and total WBC (*r* = 0.45, *P* < .01). ANC was correlated significantly with Hb level (*r* = 0.33, *P* = .04) (Fig. 1) and monocyte count (*r* = 0.26, *P* = .05).

No significant correlation was detected between anthropometric data and ANC or WBC (Table 5). Table 6 shows the calculated reference range of ANC for different groups. The reference ranges for females are lower than males, and those for Africans are lower than Asian Arabs. Table 7 shows the reference hematological vales for males and females.

**Table 5:** Correlations between ANC and other cellular parameters.

	ANC	Hb	WBC	HCT	LYMP	MONO	ESINO	BASO
ANC	1.00							
Hb	0.33*	1.00						
WBC	0.45*	−0.35	1.00					
PLT	0.07	0.02	0.14					
LYMP	0.03	0.10	0.01	0.03	1.00			
MONO	0.27	0.19	0.17	0.15	0.14	1.00		
ESINO	0.12	0.06	0.16	0.08	0.08	0.12	1.00	
BASO	0.03	0.04	0.02	0.02	−0.01	0.00	−0.02	1.00

ANC = absolute neutrophil count, BASO = basophils, ESINO = eosinophils, Hb = hemoglobin, LYMP = lymphocytes, MONO = monocytes, PLT = platelets, WBC = white blood cells.

* *P* < .01.

**Table 6:** Reference range of ANC for different groups (mean, mean + 1SD, mean + 2SD, and mean –1SD).

	Females		Males	
	Africans	Asian	Africans	Asian
Mean +2SD	6	5.94	7.06	8.03
Mean +1SD	4.25	4.27	5.13	5.9
Mean	2.5	2.6	3.2	3.77
Mean −1SD	0.75	0.93	1.27	1.64

**Table 7:** Reference hematological vales for males and females.

All females													
	Age (yr)	ANC (g/dL)	Hb (×10^9^/L)	WBC (×10^9^/L)	PLT (×10^9^/L)	HCT (%)	MCV (μm^3^)	MCHC (g/dL)	RDW (fL)	LYMP (×10^9^/L)	MONO (×10^9^/L)	ESINO (×10^9^/L)	BASO (×10^9^/L)
Mean	39.26	2.67	11.09	7.13	256.32	38.17	83.85	32.77	15.31	1.94	0.40	0.16	0.03
Standard deviation	12.20	1.94	3.57	3.82	61.64	3.15	9.89	1.49	10.65	0.57	0.20	0.14	0.03
Confidence level (95.0%)	2.09	0.34	0.62	0.84	10.70	0.54	1.70	0.26	1.83	0.10	0.03	0.02	0.00
Upper	41.36	3.01	11.70	7.97	267.02	38.71	85.55	33.03	17.14	2.04	0.44	0.18	0.04
Lower	37.17	2.34	10.47	6.29	245.63	37.63	82.15	32.51	13.48	1.84	0.37	0.13	0.03
**All males**													
	**Age (yr**)	**ANC (g/dL**)	**Hb (×10^9^/L**)	**WBC (×10^9^/L**)	**PLT (×10^9^/L**)	**HCT (%**)	**MCV (μm^3^**)	**MCHC (g/dL**)	**RDW (×10^9^/L**)	**LYMP (×10^9^/L**)	**MONO (×10^9^/L**)	**ESINO (×10^9^/L**)	**BASO (×10^9^/L**)
Mean	34.66	4.02	14.51	6.83	247.84	44.11	84.87	32.76	15.72	3.69	0.56	0.18	0.13
Standard deviation	11.38	1.20	2.07	2.53	59.99	3.89	9.80	2.41	6.50	7.41	0.30	0.14	0.02
Confidence level (95.0%)	0.91	0.91	0.17	0.20	4.79	0.31	2.38	0.19	0.52	0.59	0.02	0.01	0.17
Upper	35.57	4.92	14.67	7.03	252.63	44.42	87.25	32.95	16.24	4.28	0.58	0.19	0.29
Lower	33.75	3.11	14.34	6.63	243.05	43.80	82.48	32.57	15.20	3.10	0.53	0.17	−0.04
*P* value	.001	.001	.001	.28	.1268	.001	.2583	.5014	.63	.001	.001	.1024	.001

ANC = absolute neutrophil count, BASO = basophils, ESINO = eosinophils, Hb = hemoglobin, HCT = hematocrit, LYMP = lymphocytes, MCHC = mean corpuscular hemoglobin concentration, MCV = mean corpuscular volume, MONO = monocytes, PLT = platelets, RDW = red cell distribution width, WBC = white blood cells.

## 4. Discussion

Globally, the prevalence of neutropenia is more common in certain endemic regions. The real extent of this condition is still not fully established.^[11]^ Arabs are no exception where there is a scarcity of data on neutropenia among them. In our study, the prevalence of neutropenia (ANC < 1.5 × 10^9^/L) in 600 non-prospective blood donors (200 females and 400 males) was 10.7%. The prevalence in females was (32%) much higher compared to males (6%). The prevalence of moderate neutropenia (ANC < 1.0 × 10^9^/L) was detected in 10% of Arab females and 1.8% of Arab males.

Our data conform with the high prevalence of neutropenia reported in some countries in the Middle East and confirms the previously reported prevalence of chronic benign neutropenia (BN) in the general population of approximately 10% to15%.^[11,12]^

The findings of our study support previous few reports on the prevalence of neutropenia in the Arabian Peninsula and Arab countries in the Middle East. BN has been previously reported in Bedouin Arabs, Jordanians, Kuwaiti, Yemeni, and among some Arab tribes of Sudanese origin residing in the Middle East.^[11–16]^

In the UAE, a prospective study of a healthy indigenous population (n = 1032), found that BN was present in (10.7%) of them and (2.3%) individuals had moderate neutropenia (ANC < 1.0 × 10^9^/L). The prevalence of BN varied between 0% and 38% in the 22 tribal groups.^[11]^ However, unlike our findings, the authors did not find a statistically significant difference in the prevalence between males and females.^[17]^

In Saudi Arabia, a small study (n = 100) found that the prevalence of BN was high in the general population (up to 20%) and was more common in Saudi than the non-Saudi population. As in our study, individuals diagnosed with BN were found to have low total WBCs, differential count, lymphocyte, and monocyte counts (*P* < .01) compared to the group with normal ANC (ANC > 1.5 × 10^9^/L). Nevertheless, we reported a concordant decrease in the cell counts of monocytes and neutrophils with a significant correlation between ANC and monocyte count (*r* = 0.27) that designate a common mechanism in regulating the WBCs differentiation.^[11]^

In our study, patients with low ANC count had relatively lower Hb concentration and higher red cell distribution width (suggestive of iron deficiency) than those with normal ANC. About 30% of the females had Hb < 11 g/dL. 70% of those anemic females had ANC < 1.5 × 10^9^/L. Only 24% of the females with normal Hb levels (Hb > 11 g) had ANC < 1.5 × 10^9^/L. In addition, ANC was correlated significantly with Hb level in females (*r* = 0.33, *P* < .001). In support of our findings, patients with iron deficiency anemia had a high incidence of leukopenia and the severity of leukopenia was reported to correlate with Hb levels’ decrease in an exposure-dependent manner. This association may explain in part the higher prevalence of neutropenia in our females because of the higher prevalence of anemia.^[11,18]^

Several reports showed that the neutrophils count could be affected by ethnicity,^[11]^ where our study described a significant difference in the ANC count between African Arabs and Asian Arabs. African Arab males had significantly lower ANC and WBC counts compared to Asian Arab males. Besides, African females had significantly lower WBC counts compared to Asian Arab females. In accord with our data, in the western, African Americans, Mexican Americans, Afro-Caribbean population showed low normal limits of ANC, lower to those observed in Caucasians. Moreover, in the Arabian Peninsula, a significant difference in ANC count was found between Saudi and non-Saudi population and low ANC was reported in Yemenite Jews and some Arab populations compared to other Arabs.^[7,10,13,14,19–22]^

However, the prevalence of neutropenia in our Arab cohort was significantly higher than reported in different ethnic groups in the western populations. Hsieh et al^[7]^ reported that a prevalence of neutropenia (ANC < 1.5 × 10^9^/L) was 4.5% among “black” participants, 0.79% among “white” participants, and 0.38% among Mexican American participants. Neutrophil counts <1.0 × 10^9^/L were observed in fewer than 1% of the overall sample (0.57% in “black” participants, 0.11% in “white” participants, and 0.08% in Mexican American participants).

The results of our study and others from the Arab peninsula imply that Arabs have notably lower ANCs compared to other populations. Also, we found a significant gender difference in the ANC. The calculated reference range of ANC for different groups representing 75% of the study population (Table 6) showed that the reference ranges for females were lower than males and those for Africans were lower than Asian Arabs. With the background knowledge that our cohort included only adults and that the presence of this chronic BN has not been associated with an increased risk of infection, imply that lower cut-off values for neutrophils count is appropriate (probably of one of 1.0 × 10^9^/L). The use of a single value of ANC (1.5 × 10^9^/L for defining neutropenia in such a population would increase the unnecessary investigations for those with normal/low neutrophil count. In addition, considering the baseline values for healthy individuals with lower neutrophil count through screening may be required.^[8,14]^

Chronic BN was defined, in previous studies on Arabic tribes, as an autosomal dominant trait with an ANC < 2 × 10^9^/L.^[11]^ Genetic variance due high percentages of consanguineous marriages in the Arab world could explain the high incidence of BN where the founder gene effects the production of blood cells.^[11]^ The mass migration of many African tribes to the Arabian Peninsula could also justify the increase of neutropenia cases in Saudi Arabia for instance.^[14]^

Contrary to a previous study that found an association between higher BMI and higher WBCs and neutrophil counts in boys and girls respectively, our study did not find a significant correlation between weight or BMI and ANC or WBCs count.^[23]^

Mild to moderate neutropenia is frequent in Saudi Arabia’s southern and southwestern regions (living at high altitude). This high frequency is most likely explained by benign ethnic neutropenia.^[24,25]^ Both studies recommended that the normal neutrophil count reference range must be tailored to reflect the impact of benign ethnic neutropenia.

## 5. Limitations

There are some potential limitations due to the nature of retrospective studies; it may have included missing data which might led to bias. The small sample size might affect the generalizability of the findings.

## 6. Conclusion

Our study showed that Arabs, especially those of African origin, have relatively low ANC, with females being more affected than males. We suggest adopting a lower cutoff level for ANC in this Arab cohort to avoid unnecessary investigations for those with normal or low neutrophil count. Further studies are required to understand the impact of ethnicity on different blood values and implementing personalized reference ranges of laboratory tests is highly recommended.

## Author contributions

**Conceptualization:** Mohamed A. Yassin.

**Data curation:** Mohamed A. Yassin, Ashraf T. Soliman, Saloua M. Hmissi, Mohammad A.J. Abdulla, Ans A. Alamami, Mahmood B. Aldapt, Aasir M. Suliman, Ezzeddin A. Ibrahim, Mouhand F.H. Mohamed, Shehab F. Mohamed, Abdulqadir J. Nashwan.

**Formal analysis:** Prem Chandra.

**Methodology:** Mohamed A. Yassin, Ashraf T. Soliman, Saloua M. Hmissi, Mohammad A.J. Abdulla, Ans A. Alamami, Mahmood B. Aldapt, Aasir M. Suliman, Ezzeddin A. Ibrahim, Mouhand F.H. Mohamed, Shehab F. Mohamed, Abdulqadir J. Nashwan.

**Writing – original draft:** Mohamed A. Yassin, Ashraf T. Soliman, Saloua M. Hmissi, Mohammad A.J. Abdulla, Ans A. Alamami, Mahmood B. Aldapt, Aasir M. Suliman, Ezzeddin A. Ibrahim, Mouhand F.H. Mohamed, Shehab F. Mohamed, Prem Chandra, Abdulqadir J. Nashwan.

**Writing – review & editing:** Mohamed A. Yassin, Ashraf T. Soliman, Saloua M. Hmissi, Mohammad A.J. Abdulla, Maya Itani, Ans A. Alamami, Mahmood B. Aldapt, Aasir M. Suliman, Ezzeddin A. Ibrahim, Mouhand F.H. Mohamed, Waail Rozi, Shehab F. Mohamed, Prem Chandra, Abdulqadir J. Nashwan.

All authors read and approved the final manuscript.
